# A randomised controlled trial of the Neuro Emotional Technique (NET) for childhood Attention Deficit Hyperactivity Disorder (ADHD): a protocol

**DOI:** 10.1186/1745-6215-10-6

**Published:** 2009-01-27

**Authors:** Fay Karpouzis, Henry Pollard, Rod Bonello

**Affiliations:** 1 Department of Health and Chiropractic, Macquarie University, Sydney, 2109, Australia; 2 Macquarie Injury Management Group (MIMG), Macquarie University, Sydney, 2109, Australia

## Abstract

**Background:**

An abundance of literature is dedicated to research for the treatment of Attention Deficit Hyperactivity Disorder (ADHD). Most, is in the area of pharmacological therapies with less emphasis in psychotherapy and psychosocial interventions and even less in the area of complementary and alternative medicine (CAM).

The use of CAM has increased over the years, especially for developmental and behavioral disorders, such as ADHD. 60–65% of parents with children with ADHD have used CAM. Medical evidence supports a multidisciplinary approach (i.e. pharmacological and psychosocial) for the best clinical outcomes. The Neuro Emotional Technique (NET), a branch of Chiropractic, was designed to address the biopsychosocial aspects of acute and chronic conditions including non-musculoskeletal conditions. Anecdotally, it has been suggested that ADHD may be managed effectively by NET.

**Design/methods:**

A placebo controlled, double blind randomised clinical trial was designed to assess the effectiveness of NET on a cohort of children with medically diagnosed ADHD.

Children aged 5–12 years who met the inclusion criteria were randomised to one of three groups. The control group continued on their existing medical regimen and the intervention and placebo groups had the addition of the NET and sham NET protocols added to their regimen respectively. These two groups attended a clinical facility twice a week for the first month and then once a month for six months.

The Conners' Parent and Teacher Rating Scales (CRS) were used at the start of the study to establish baseline data and then in one month and in seven months time, at the conclusion of the study. The primary outcome measures chosen were the Conners' ADHD Index and Conners' Global Index. The secondary outcome measures chosen were the DSM-IV: Inattentive, the DSM-IV:Hyperactive-Impulsive, and the DSM-IV:Total subscales from the Conners' Rating Scales, monitoring changes in inattention, hyperactivity and impulsivity.

Calculations for the sample size were set with a significance level of 0.05 and the power of 80%, yielding a sample size of 93.

**Discussion:**

The present study should provide information as to whether the addition of NET to an existing medical regimen can improve outcomes for children with ADHD.

**Trial registration:**

Australian New Zealand Clinical Trial Registration Number: ANZCTRN 012606000332527

## Background

There is an abundance of literature dedicated to research for the treatment of Attention Deficit Hyperactivity Disorder (ADHD) [1-5]. Most of this research is in the area of pharmacological therapies with less emphasis in psychotherapy and psychosocial interventions [6-8] and even less in the area of complementary and alternative medicine (CAM) [9,10].

The use of CAM therapies, has increased over the years, especially for the developmental and behavioral disorders, such as ADHD[9,11-14]. Approximately 60–65% of parents with children diagnosed with ADHD have used CAM therapies [9,11,13,15,16]. Despite varied definitions of what constitutes a CAM therapy, most definitions of CAM include chiropractic. Whilst there is a great deal of research on spinal manipulation performed by chiropractors, there are very few studies to substantiate  the effect chiropractic treatment has on children with ADHD [17,18]. Most published articles are of case studies [19-21] case series [18,22,23] and small cohort studies with sample sizes ranging up to N = 7 [24,25]. Of these, only three are from peer-reviewed journals [19,20,24] regardless, all of these provide a low level of evidence without the follow-up of randomised controlled trials (RCT) to substantiate findings.

Medical evidence supports a multidisciplinary approach (i.e. pharmacological and psychosocial) for children and adolescents diagnosed with ADHD for the best clinical outcomes [1,3,7,8,26-32]. In fact, certain studies have shown that the multidisciplinary approach produces additional benefits in that there is greater satisfaction amongst parents and teachers as this approach leads to lower medication dosages for these children [29,33]. It is well established that ADHD is a multi-factorial disorder, having both psychological and social components, that can have negative outcomes if not diagnosed and managed effectively [1,7,8,26,34-36].

The best clinical outcomes are produced when an integrated biopsychosocial approach is employed for children with ADHD [27,37-39]. The Neuro Emotional Technique (NET), a branch of Chiropractic, was designed to address the biopsychosocial aspects of acute and chronic conditions including non-musculoskeletal conditions [40].

Evidence suggests that 10–30% of children with ADHD who have been prescribed stimulant medications, do not show clinically significant outcomes, and others experience side-effects and need to discontinue their medications [26,31,32,41-43].

The most common side effects of stimulant medications are appetite suppression, weight loss, sleep disturbances, irritability, stomach aches, headaches, rashes, and occasionally the development or aggravation of tics [3,6,26,29,38,41,43,44].

For these children, additional strategies need to be implemented in order to achieve a successful outcome [1,42]. Parents with children diagnosed with ADHD who have had less than satisfactory outcomes with conventional management are presenting to chiropractors looking for alternative interventions. Anecdotally, it has been suggested that ADHD may be managed effectively by NET. There are published case studies on the use of NET for conditions such as hypothyroidism [45], anovulation infertility [46], polycystic ovary syndrome [47], separation anxiety disorder [48], a case series on cancer-related traumatic stress symptoms [49] and a RCT for trigger point sensitivity in chronic neck pain sufferers [50]. However, there is no validation at this time to substantiate the use of NET for children with ADHD. As a result, the application of NET to a cohort of children with medically diagnosed ADHD is being researched. It is important to research an approach being used with a clinic population for which it was not previously designed for nor tested on. The purpose of this paper is to outline the methodology for the RCT employing the CONSORT [51,52] checklist in its design.

### Objectives

The objectives of this research are to:

1. To determine whether the addition of the Neuro Emotional Technique (a new biopsychosocial intervention), to an existing medical regimen can improve clinical outcomes (i.e. reduce inattention, hyperactivity and impulsivity) for children with medically diagnosed ADHD.

2. Furthermore, to determine the responses in the short term (i.e. one month) and medium term (seven months).

## Methods

### Design

Ethics approved, registered, interdisciplinary, placebo controlled, double blind, randomized clinical trial.

### Settings

Four private clinics in Sydney, Australia.

### Care providers

Four chiropractors, three male and one female volunteered their time and services to administer interventions to the participants of the study at no cost. The chiropractors had varied tertiary qualifications: one had Bachelor of Medical Sciences and a Master of Chiropractic; two had a Bachelor of Science and a Master of Chiropractic and one had Bachelor of Science and a Graduate Diploma of Chiropractic. All of the chiropractors had undergone the four NET training workshops over a two year period, and had passed the certification exams in both theory and practice, and a thus were qualified as NET certified practitioners. These practitioners were chosen on the basis that they were experts in the field, were certified and agreed to abide by the study protocols.

### Participants

Parents were initially screened on the phone, and their children were included in the study if inclusion criteria were met. Inclusion criteria comprised: children aged between 5–12 years; children with a medical diagnosis of ADHD for minimum of 2 months, diagnosed by Pediatrician, Psychiatrist or Clinical Psychologist; informed verbal and written consent from parents and participants; children currently undergoing any interventions (pharmacological, psychosocial therapy, educational, occupational therapies etc) or children with the diagnosis of ADHD who have not undergone any treatment; and children who presented with the following comorbid disorders – Conduct Disorder, Oppositional Defiant Disorder, Learning Disabilities, and Anxiety Disorders. Any children who did not meet the inclusion criteria, or who met the exclusion criteria were excluded from the study. Exclusion criteria comprised: A diagnosis of Language difficulties, Mood Disorders (eg. Depressive Disorder or Bipolar Disorder), Communication Disorder, mutism, Pervasive Developmental Disorder (eg. Autism or Asperger's Disorder), Psychosis, profound deafness and other physical or mental disabilities that would prevent participants interacting physically and verbally with the care provider during the consultation; any changes in medications during the course of the study, and any child or parent who had previously undergone any NET treatment.

### Measures

The Diagnostic and Statistical Manual of Mental Disorders ADHD diagnostic criteria [53,54] were used by the participants' Pediatricians and Clinical Psychologists to establish a diagnosis. The reliable and validated Conners' Parent Rating Scales-Revised Long Version (CPRS-R:L) and Conners' Teacher Rating Scales-Revised Long Version (CTRS-R:L) [55-59] were used pre and post intervention.

The ratings scales were administered pre-intervention to establish baseline data, and were used as outcome assessments to measure the intervention effects at the end of the first month and at the conclusion of the study i.e. in seven months. Of the 14 subscales described in the CPRS-R:L and the 13 subscales described in the CTRS-R:L, two were chosen as primary outcome measures (POMs) and three were chosen as secondary outcome measures (SOMs). Primary outcome measures included Conners' ADHD Index and Conners' Global Index Total subscales H and K respectively. The secondary outcome measures included DSM-IV:Inattentive, DSM-IV:Hyperactive-Impulsive and DSM-IV:Total, subscales L, M, and N respectively.

#### Primary Outcome Measures

The Conners' ADHD Index is used to distinguish ADHD children from non-clinical children, and is considered a good screening tool [55,59]. The Conners' Global Index (CGI) is valuable in assessing general psychopathology and for monitoring treatment effectiveness and changes over time. It is sensitive to treatment changes for repeated measures [55,56]. It reflects general behavioral problems, including hyperactivity and attentional difficulties.

#### Secondary Outcome Measures

The DSM-IV:Inattentive subscale was chosen as it has a correlation with the DSM-IV diagnostic criteria for the Inattentive Subtype of ADHD, and focuses on the symptom of inattention. The DSM-IV:Hyperactive-Impulsive subscale was chosen as it has a correlation with the DSM-IV diagnostic criteria for the Hyperactive-Impulsive Subtype of ADHD, and focuses on the symptoms of hyperactivity and impulsivity. The DSM-IV:Total subscale was chosen as it has a correlation with the DSM-IV diagnostic criteria for the Combined Subtype of ADHD, and focuses on all core features of ADHD (i.e. inattention, hyperactivity and impulsivity) [55].

### Treatment allocation

Eligible participants were randomly allocated to one of three groups A, B, & C (i.e. group A-sham, group B-treatment, and group C-control). The "QuickCalcs" program from the GraphPad Software [60] was used to randomly sequence 150 numbers (repeated 1×) into the three groups.

Each number and its randomised letter (A, B, or C) were placed in an opaque envelope. Each envelope was numbered consecutively from 1–150.

As each parent called to have their child screened they were allocated the next consecutive number once they were eligible, so that eligible participant no. 1 was allocated to envelope no. 1, etc. The screener was unaware of the sequence of group allocation at the time of screening, hence allocation was concealed at time of recruiting.

### Blinding

Participants were blinded as to which intervention group they were allocated to, in an attempt to control for the Hawthorne Effect. Parents and teachers were blinded to which intervention group the children were allocated to, in an attempt to minimize rater bias when completing rating scales i.e. CPRS-R:L and CTRS-R:L. The registered Psychologist and the Clinical Psychologist were blinded as to which group participants were allocated to, in order to minimize bias when scoring and interpreting the data from the CPRS-R:L and CTRS-R:L.

The NET practitioners were not blinded in this study as they needed to know which participants were receiving which intervention, i.e. treatment versus sham protocol. The parents of the control group were also aware that their children were in the control group awaiting treatment at the completion of the study.

### Sample size justification

Calculations for the sample size were set with a significance level of 0.05 and the power of 80%.

As there are no prior studies conducted in the area of chiropractic or NET for an ADHD cohort, for power analysis purposes a SD of 6.7 was adopted based on data from Shortt [61], yielding a sample size of 63. The value of 6.7 for power testing is supported by findings from a paper on the meta-analysis of RCTs in ADHD responses to atomoxetine using the CRS [62]. This study revealed an average SD score of 6.7, which is consistent with the SD found in the Shortt data [61]. In a meta-analysis of psychotherapy dropouts the mean attrition rate was 46.86% [63,64]. Based on this drop-out rate it was prudent to increase the sample size by this figure, increasing it from 63 to n = 93.

### Recruitment

Recruitment for the study commenced in July 2006 and the first participant was enrolled in August 2006 and the last participant completed the study in March 2008. Each participant that qualified and was enrolled into the study and had their own start date, hence the staggered starts and as a result the study took 17 months to complete.

### Withdrawals

As part of the phone screening and the informed consent forms the parents of the participants were advised that they were free to withdraw their child/children from the study at any stage without any consequences.

### Protocol

Group A, the sham group, continued with their existing program and the sham protocol was added to their regimen. Group B, the treatment group, continued with their existing program and the NET protocol was added to their regimen. The participants in groups A and B attended a clinical facility for the first month and received eight treatments (two treatments per week) for approximately 15–20 minute sessions. Then, they were scheduled for another six treatments over the next six months (i.e. one treatment per month). Group C, the control group, consisted of children who continued with their existing ADHD treatment regimen as prescribed by their pediatrician, clinical psychologist and/or medical doctor.

### Neuro Emotional Technique (NET) protocol

#### Definition of NET

NET is a methodology of finding and removing Neuro Emotional Complexes (NECs). A NEC is defined as a subjective maladaptation syndrome adopted by the organism in response to a real or perceived threat to any aspect of its survival [40]. NET has been described as a treatment designed to address negative distressing stimuli, by removing these patterns by accessing the nervous system via stimulation of the spine.

**Figure 1:**
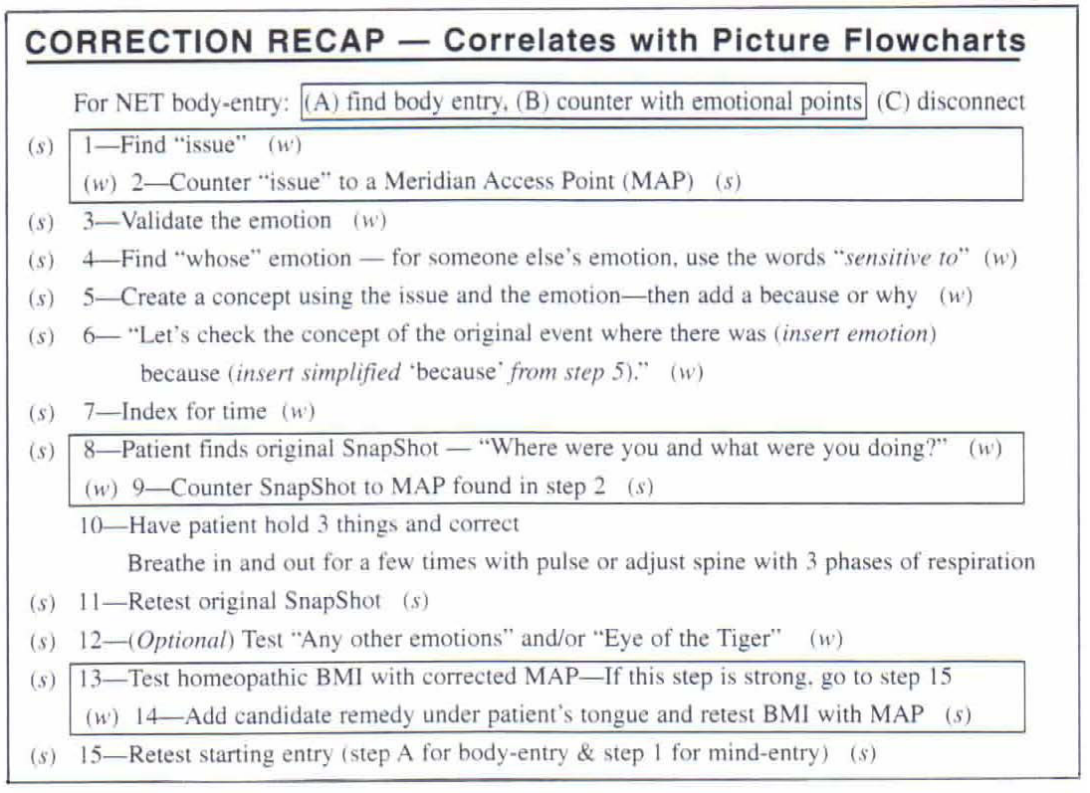
**15-step NET protocol**.

Walker [40] described NET as a 15 step (Figure 1), multi-modal system that integrates the principles of several health modalities, including cognitive behavioural psychology principles, traditional Chinese pulse assessment, acupuncture theory and the feedback technique called muscle testing.

Muscle testing is non-invasive system of evaluating the function of the body [65]. NET protocol uses muscle testing throughout the procedure as an indicator for physiologic reactivity to a protocol of statements designed to challenge the cognitive recall of stimuli under contemplation [66].

NET is a system that evaluates structural, emotional, and chemical (i.e. toxic and nutritional) aspects of a patient's health and wellbeing using the manual muscle testing procedure as a diagnostic guide. Emphasis must be made that for the purpose of this study, the emotional aspects were the only issues addressed, investigating whether ones "emotional reality" affected one's mental wellbeing and behaviour patterns.

#### NET protocol

Firstly, the participants were evaluated for a healthy deltoid muscle, capable of resisting the testing pressure of the practitioner. The participants were seated, and asked to raise their arm 90 degrees to their body, keeping their elbow straight. Pressure was applied to the participant's wrist, whilst they were asked to "hold strong", (i.e. resist pressure). A practice trial was conducted to familiarize the participants with muscle testing procedure (MTP). This MTP has shown good inter-examiner reliability [67].

The participants were asked to make the following statement, "I'm OK, keeping still."

If MTP to this statement tested weak, it is said that the participant is non-congruent with that concept.

**Figure 2:**
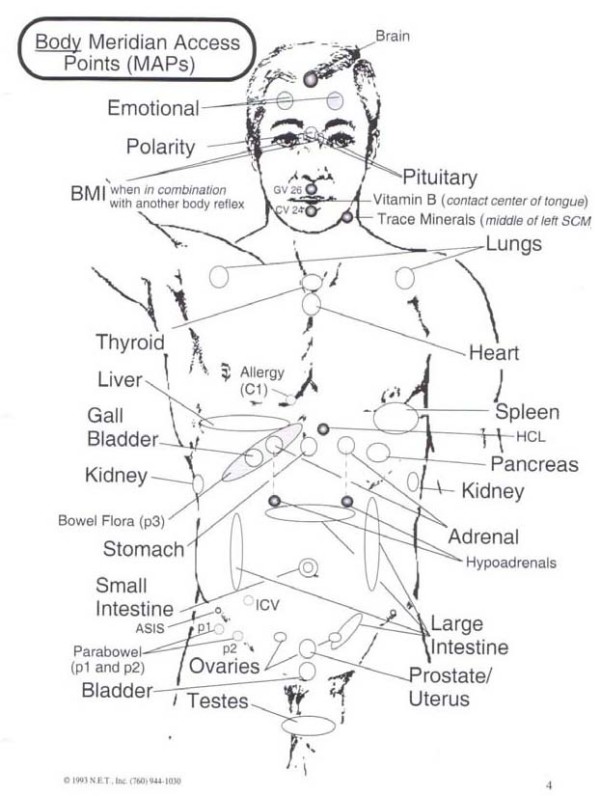
**NET Body Meridian Access Points (MAPs)**.

**Figure 3:**
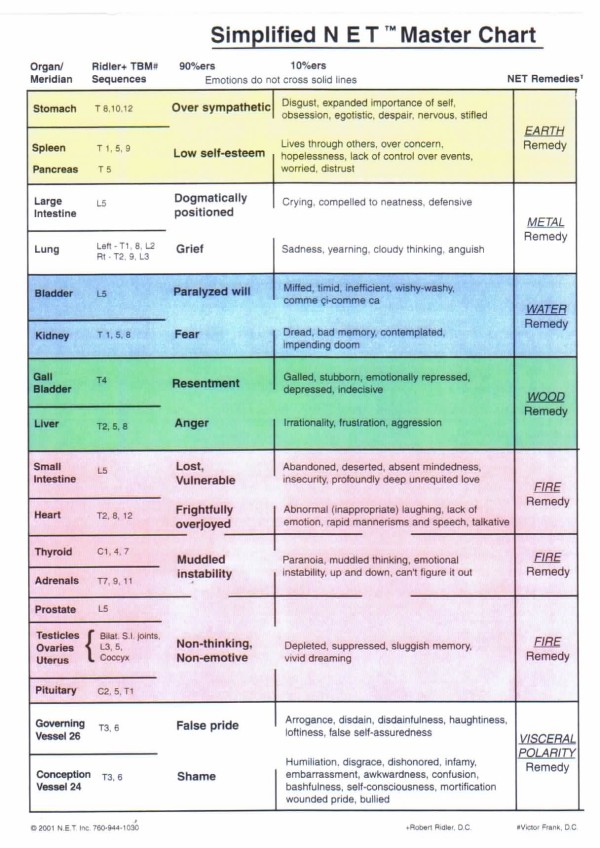
**Simplified Master NET Chart outlining Meridians/Organs; Subluxation sequences; Emotions; NET Remedies**.

Next, the participants were asked to continually repeat that statement whilst the practitioner continued the MTP with one hand and with the other hand palpated the different meridian access points (MAPs) on the participant's body. Each MAP is considered to be a specific skin point which, based on acupuncture theory is associated with certain emotions [68]. (Figure 2 and 3)

When the participants tested strong to the statement and a MAP, it was considered that there was an emotional component related to their problem.

The practitioner continued to explore the psychosomatic connection by making a series of statements. Using the MTP the concept of an "original event" was being investigated. The current "emotion" was related to a past event by the asking of "when, where, and who" questions. This was done to uncover why there appeared to be retained conditioned response to a previous stressful event.

The untested theory of NET suggests that the stimuli associated with the original event become associated via a process of Pavlovian conditioning, and are then reproduced through a process of repetition compulsion.

The participants were provided a statement that connected past and present events relating to their "emotion". If the MTP using this statement, tested weak, it indicated that the problem had been recalled.

Next, the participants were asked to contemplate the original event and associated feelings in the conscious mind, and were given kinaesthetic stimulation of spinal segments. The conscious recall of past events with the association of normalised stimuli is theorised to condition the original event with positive stimuli. This is believed to break the cycle of the conditioned (learnt) response by reactivating the original negative event and it's ongoing repetition and pairing it with a competing somatic stimulus.

Lastly, the practitioner retested the participant by using MTP to check the original statement "I'm OK keeping still". When the MTP tests strong, this indicates the participant is now congruent with that statement.

This procedure was repeated using 16 different referential statements. During the first month of treatment two referential statements per visit were used and tested. If the MTP tested weak, implying the participant was not congruent with the concept in that particular statement, then the NET protocol ensued. Please see Table 1 for a complete list of statements used. The statements were designed by the primary investigator in consultation with an expert NET practitioner and the study supervisors using the DSM-IV criteria which are used to diagnose ADHD and coupled them with the standard 15 step NET protocol (Figure 1), designed by Walker [40]. Another explanation of this protocol is described in a study published by Bablis and colleagues [50].

**Table 1:** List of referential statements used in NET protocol for treatment group B.

**Referential statements**	**Muscle testing procedure (MTP)**	
	**Non-congruent (weak muscle)**	**Congruent (strong muscle)**

I'm OK having ADHD		

I'm OK keeping still		

I'm OK listening to authority		

I'm OK waiting my turn		

I'm OK thinking one thing at a time		

I'm OK thinking before doing		

I'm OK remembering what I'm told		

I'm OK finishing what I start		

I'm OK doing homework		

I'm OK doing class work		

I'm OK listening to the teacher		

I'm OK doing school work		

I'm OK thinking before talking		

I'm OK staying in my seat		

I'm OK having quiet time		

I'm OK going to sleep at bedtime		

### Sham NET protocol

The sham protocol was designed to mimic the real NET treatment protocol, however without the therapeutic benefits. The sham protocol incorporated the MTP throughout the procedure, with different sets of statements and tapping on the back. The set of statements used were not related to the treatment protocol, but instead used for testing of congruent and non-congruent reactions to statements like, " my name is John" and then "my name is Robert" i.e. the child's name first then another name. See Table 2 for a complete list of the first Sham protocol. In total eight different sham protocols were designed with different lists of referential statements.

**Table 2:** List of referential statements used in sham protocol for sham group A.

**Referential statements for sham**	**Muscle Test**	
	**Weak**	**Strong**

My name is .......... (insert child's name)		

My name is .......... (make up wrong name)		

My age is ............ years (insert child's age)		

My age is ............ years (insert wrong age)		

I live in ............... (insert child's suburb)		

I live in .............. (insert wrong suburb)		

Today is ............. (insert the name of today)		

Today is ............. (insert the wrong day)		

My hair colour is .... (insert colour of child's hair)		

My hair colour is .... (insert wrong colour of child's hair)		

My eye colour is .... (insert colour of child's eyes)		

My eye colour is .... (insert wrong colour of child's eyes)		

I am in ............... class at school (insert child's class)		

I am in ............... class at school (insert wrong class)		

I go to ................ (insert name of child's school)		

I go to ................ (insert name of wrong school)		

My teachers name is .... (insert name of teacher)		

My teachers name is ... (insert wrong name of teacher)		

My favourite sport is ... (insert name of favourite sport)		

My favourite sport is ... (insert name of wrong sport)		

My favourite subject at school is ... (insert favourite subject)		

My favourite subject at school is ... (insert wrong subject)		

My worst subject at school is ... (insert worst subject)		

My worst subject at school is ... (insert different subject)		

My best friend is ... (insert name of best friend)		

My best friend is ... (insert wrong name)		

### Risks

As there are no known side effects and there have not been any reports of adverse reactions to the NET protocol, we do not anticipate that any participants will be exposed to any unnecessary risks. However, in the event that a child becomes emotionally distressed during the protocol, the procedure will be terminated. Support counselling will be offered by qualified psychotherapists and counsellors for the child at no expense to the parents. These professionals have all been approved for the study by the university Ethics Committee and have also undergone background screening and have been approved to work with children.

### Ethics review

The trial protocol was reviewed by the Macquarie University Human Ethics Committee (Sydney, Australia) and was granted Human Ethics Approval (HE26AUG2005-M04261). As a prerequisite to conducting research with children, the Ethics committee required all those involved with the study undergo background screening as part of the "Working With Children Check". All those involved in the study were approved and ethics was granted.

Furthermore, as part of this study, an application was made to the Department of Education and Training and a State Education Research Approval was granted (SERAP #06.350). This enabled the researchers to send questionnaires to the teachers of the participants of the study.

### Informed consent

All of the parents of the participants were issued with two copies of the informed consent forms (one to keep and one to return to the university). The informed consent form conveyed the following information: the names of the chief investigator, the supervisors and the four practitioners administering the interventions; the name of the university and the department conducting the research; a brief description of the control, treatment and sham groups; the duration of the study; the questionnaires used; the Ethics approval and contact details of the Ethics officer; the right to withdraw their child at any time; in the event a child becomes distressed the back-up counselling services available; the involvement of teachers; and the fact that the results of the study will be disseminated via conferences and publications whilst maintaining the children's privacy.

### Analysis

Demographic and baseline characteristics of participant variables will be compared between the three randomised groups using the Restricted Maximum Likelihood (REML) in GenStat 10^th ^Edition [69].

The primary analysis will compare outcomes for each primary and secondary outcome measure between the three groups (treatment, sham and control) using the Restricted Maximum Likelihood (REML) in GenStat 10^th ^Edition [69], to estimate treatment effect with 95% CI. REML is a method for fitting mixed linear models (the models for ANOVA being special cases) which can produce unbiased (or less biased) estimates of variance and covariance parameters. This is a relatively new and powerful technique that allows for data sets that are unbalanced, have missing values, have treatments with unequal variances and/or have correlated error terms [70].

An intention-to-treat (ITT) analysis will be undertaken of the one month and seven month data from the CPRS-R:L and CTRS-R:L for the participants who commenced the study but did not complete.

## Discussion

We have a presented the design and the protocol for the RCT of NET for childhood ADHD. The participants of this study will have NET added to their existing medical regimen (pharmacologic and/or psychosocial) and will be monitored for changes in their outcomes (i.e. inattention, hyperactivity and impulsivity) in the short term (one month) and medium term (7 months). Completion of this trial will help provide the answer to the question "does chiropractic (NET) have a role to play in the management of children with ADHD?" Results of this RCT will be disseminated at the completion of the study.

## Abbreviations

NET: Neuro Emotional Technique; ADHD: Attention Deficit Hyperactivity Disorder; CAM: Complementary and Alternative Medicine; CONSORT: Consolidated Standard of Reporting Trials; DSM-IV: Diagnostic and Statistical Manual of Mental Disorders 4^th ^Edition; CRS: Conners' rating Scales; CPRS-R:L: Conners' Parent Rating Scale Revised Long Version; CTRS-R:L: Conners' Teacher Rating Scale Revised Long Version; POMs: Primary Outcome Measures; SOMs: Secondary Outcome Measures; CGI: Conners' Global Index; CBT: Cognitive Behavioral Therapy; NECs: Neuro Emotional Complex; MTP: Muscle Testing Procedure; SERAP: State Education Research Approval; ANOVA: Analysis of Variance; ITT: Intention-to-Treat Analysis; CI: Confidence Intervals; RCT: Randomized Controlled Trial.

## Competing interests

No funding was received for this study or for the preparation of this manuscript.

The chief investigator FK and secondary supervisor RB declare that they have no competing interests.

The primary supervisor HP is a part-time employee of the ONE (Our Net Effect) Foundation, which is the research arm of NET and a non-profit organisation. The organisations interests relate to establishing natural healing as standardised care through NET research, education and public service.

## Authors' contributions

FK conceived the research project.

FK, HP, and RB designed the study.

FK designed and tailored the protocol for the ADHD cohort.

All authors contributed to the writing of the manuscript.

All authors read and approved the final manuscript.
